# Hypoxia-Inducible Factor 1-Alpha and Glucose Metabolism during Cardiac Remodeling Progression from Hypertrophy to Heart Failure

**DOI:** 10.3390/ijms24076201

**Published:** 2023-03-25

**Authors:** Paula Grippa Sant’Ana, Loreta Casquel de Tomasi, Gilson Masahiro Murata, Danielle Fernandes Vileigas, Gustavo Augusto Ferreira Mota, Sérgio Luiz Borges de Souza, Vitor Loureiro Silva, Livia Paschoalino de Campos, Katashi Okoshi, Carlos Roberto Padovani, Antonio Carlos Cicogna

**Affiliations:** 1 Department of Internal Medicine, Botucatu Medical School, São Paulo State University (UNESP), Botucatu 18618-687, Brazil; 2 Laboratory of Medical Investigation (LIM-29), Division of Nephrology, University of São Paulo Medical School, São Paulo 01246-903, Brazil; 3 Department of Biostatistics, Institute of Biosciences, São Paulo State University (UNESP), Botucatu 18618-689, Brazil

**Keywords:** aortic stenosis, cardiac remodeling, HIF-1α, glucose metabolism, rats

## Abstract

In pathological cardiac hypertrophy, the heart is more dependent on glucose than fatty acids. This shift in energy metabolism occurs due to several factors, including the oxygen deficit, which activates hypoxia-inducible factor-1α (HIF-1α), a critical molecule related to glucose metabolism. However, there are gaps regarding the behavior of key proteins in the glycolytic pathway and HIF-1α during the transition from hypertrophy to heart failure (HF). This study assesses the hypothesis that there is an early change and enhancement of HIF-1α and the glycolytic pathway, as well as an association between them during cardiac remodeling. Sham and aortic stenosis Wistar rats were analyzed at 2, 6, and 18 weeks and in HF (*n* = 10–18). Cardiac structure and function were investigated by echocardiogram. Myocardial glycolysis, the aerobic and anaerobic pathways and glycogen were analyzed by enzymatic assay, Western blot, and enzyme-linked immunosorbent assay (ELISA). The following were observed: increased left ventricular hypertrophy; early diastolic function change and severe systolic and diastolic dysfunction in HF; increased HIF-1α in the 2nd week and in HF; precocious alteration and intensification of glycolysis with a shift to anaerobic metabolism from the 6th week onwards; association between HIF-1α, glycolysis, and the anaerobic pathway. Our hypothesis was confirmed as there was an early change and intensification in glucose metabolism, alteration in HIF-1α, and an association between data during the progression from hypertrophy to heart failure.

## 1. Introduction

The heart can meet energy demands using various substrates, such as glucose, lactate, fatty acids, amino acids, and ketone bodies [1,2]. Around 40–70% of the energy needed during the contraction and relaxation processes in the adult heart is usually obtained from fatty acids, with glucose contributing about 20–30% [2,3].

In pathological cardiac hypertrophy, the metabolism is reprogrammed with greater dependence on glucose to the detriment of fatty acids so that the heart reverts to the fetal metabolic condition [1,2]. The mechanisms involved in switching the substrates are not sufficiently clarified, and the classic explanation for this imbalance requires oxygen (O_2_) deficit [4].

In response to the lack of O_2_ and adenosine triphosphate (ATP) [5], protein expression changes occur in different cellular processes [5,6,7,8] to increase tissue O_2_ and ATP availability to ensure cell maintenance and survival [5,6]. One of the main factors involved in these events is attributed to hypoxia-inducible factor-1α (HIF-1α), a transcriptional factor responsible for activating hundreds of genes, including those associated with glucose metabolism [9,10,11].

HIF-1α intensifies the glycolytic pathway during the cardiac remodeling (CR) process [12]. Although there is an extensive body of literature on glucose metabolism in different types of heart disease [13,14,15,16,17,18,19], showing an increased reliance on glucose with an overall reduced oxidative metabolism, i.e., a reappearance of the fetal metabolic pattern, there are gaps concerning the involvement of HIF-1α and glucose metabolism during CR progression. This study aimed to evaluate the hypothesis that there is an early change and intensification of HIF-1α, glucose metabolism, and the association between these variables during the progression from hypertrophy to heart failure (HF). Knowing the initial moment of O_2_ deficit and metabolic alterations will shed light on the molecular mechanisms underlying CR progression and assist in the development of possible therapeutic strategies in treating heart disease due to chronic pressure overload.

## 2. Results

### 2.1. Clinical-Pathological Signs of Heart Failure

All rats in the AoS_HF_ group presented altered breathing patterns, ascites, pleural effusion, right ventricular (RV) hypertrophy, and left atrial thrombus. The left atrium showed a large thrombus in 80% of the rats and a moderate thrombus in 20%; RV hypertrophy was marked in 90% and moderate in 10%. The Sham_HF_ group showed no clinical or pathological signs of HF (Table 1).

### 2.2. Body and Lung Weights and Post Mortem Cardiac Morphology

**Figure 1:**
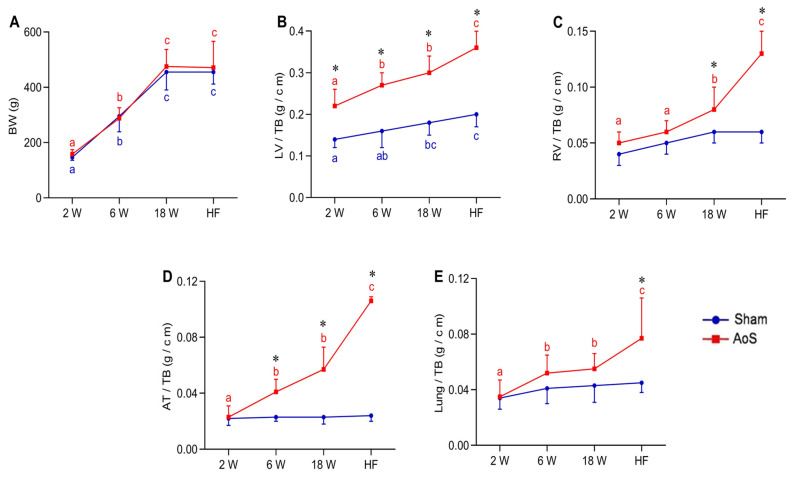
Bodyweight and post mortem cardiac morphology at different euthanasia moments (2, 6, 18 weeks [W] and heart failure [HF]). Sham: operated control (blue); AoS: supravalvar aortic stenosis (red). (**A**) BW: body weight. (**B**) LV/TB: left ventricle corrected by tibia (TB). (**C**) RV/TB: right ventricle corrected by TB; (**D**) AT/TB: atrium corrected by TB; (**E**) Lung/TB: lung corrected by TB. Data are expressed as mean ± SD and analyzed by ANOVA and Tukey. *p* < 0.05. * AoS vs. respective Sham. Distinct letters signify statistical difference between moments (*n* = 12–18 animals for each group).

Body weight increased over time and stabilized after the 18th week in both the Sham and Aos groups (Figure 1A). Left ventricle corrected by tibia (LV/TB) in Aos_HF_ was higher than all other AoS groups; it was more significant in Aos_18_ and AoS_6_ than AoS_2_. This variable was also higher in Sham_HF_ than Sham_6_ and Sham_2_ and more prominent in Sham_18_ than Sham_2_. LV/TB was higher in AoS groups compared to their respective Shams (Figure 1B).

RV/TB was higher in Aos_HF_ than the other AoS groups; it was more significant in Aos_18_ and AoS_6_ than AoS_2_. RV corrected by tibia (RV/TB) was higher in AoS_HF_ and AoS_18_ groups compared to their respective Shams (Figure 1C). Atrium corrected by TB (AT/TB) and lung corrected by TB (Lung/TB) were more prominent in Aos_HF_ than the other AoS groups; these variables were also higher in Aos_18_ and AoS_6_ than AoS_2_. AT/TB were higher in AoS_HF_, AoS_18_, and AoS_6_ compared to their respective Shams, while Lung/TB was more prominent in AoS_HF_ than Sham_HF_ (Figure 1D,E). These data indicate a dysfunctional hypertrophied heart that was accentuated in the HF phase.

### 2.3. Heart Rate, Structural and Functional Echocardiographic Data

**Figure 2:**
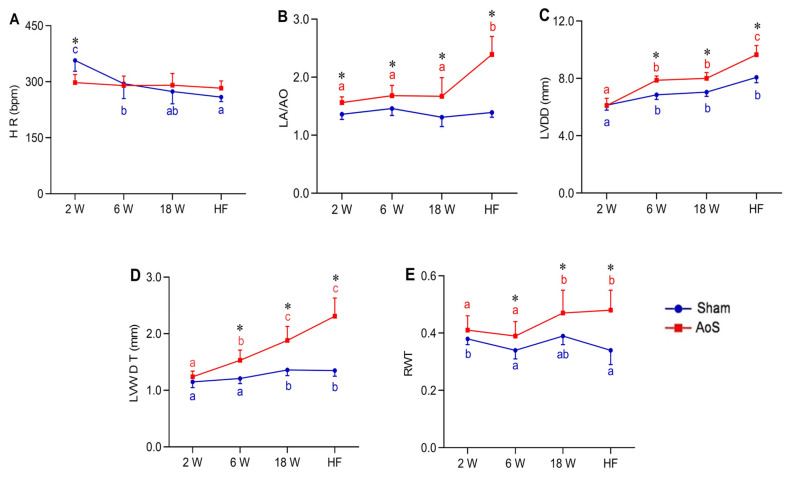
Heart rate and structural echocardiographic data at different euthanasia moments (2, 6, 18 weeks [W] and heart failure [HF]). Sham: operated control (blue); AoS: supravalvar aortic stenosis (red). (**A**) HR: heart rate; (**B**) LA/AO: left atrial diameter (LA) to aortic diameter (AO) ratio; (**C**) LVDD: left ventricle (LV) diastolic diameter; (**D**) LVWDT: LV posterior wall diastolic thickness; (**E**) RWT: LV relative wall thickness. Data are expressed as mean ± SD and analyzed by ANOVA and Tukey. *p* < 0.05. * AoS vs. respective Sham. Distinct letters signify statistical difference between moments (*n* = 12–18 animals for each group).

Heart rate (HR) was more significant in Sham_2_ than the other control groups; it was higher in Sham_6_ than Sham_HF_. HR in AoS_2_ animals was lower than in Sham_2_ (Figure 2A). Left atrium diameter normalized to aortic diameter (LA/AO) ratio was more significant in AoS**_HF_** than the other three AoS groups; this ratio was higher in all AoS groups in relation to their respective Sham group (Figure 2B). Left ventricle diastolic diameter (LVDD) was higher in AoS_HF_ than the other AoS groups; this variable was higher in AoS_6_ and AoS_18_ than AoS_2_. It was also lower in Sham_2_ than the other Sham groups; LVDD was higher in AoS_6_, AoS_18_, and AoS_HF_ in relation to their respective controls (Figure 2C). Left ventricle posterior wall diastolic thickness (LVWDT) was higher in AoS_HF_ and AoS_18_ than AoS_6_ and AoS_2_; it was also higher in AoS_6_ than AoS_2_. In Sham_HF_ and Sham_18_, it was higher than in Sham_6_ and Sham_2_. LVWDT was higher in AoS_6_, AoS_18_, and AoS_HF_ in relation to their respective Sham groups (Figure 2D). Left ventricle relative wall thickness (RWT) was higher in AoS_18_ and AoS_HF_ than AoS_2_ and AoS_6_; it was also higher in Sham_2_ than Sham_6_. RWT was higher in AoS_6_, AoS_18_, and AoS_HF_ than their respective Sham groups (Figure 2E). Echocardiographic data show hypertrophy development over time in animals who underwent AoS surgery.

**Figure 3:**
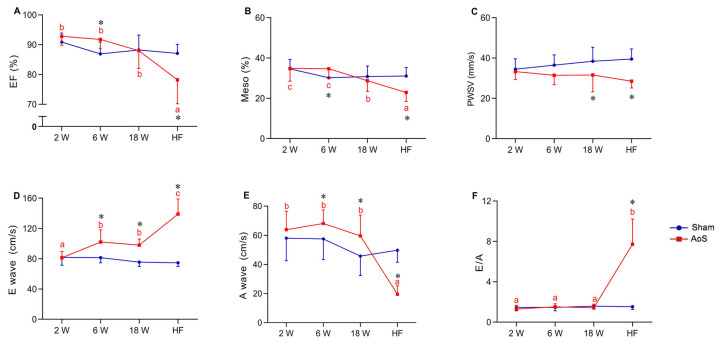
Functional echocardiographic data at different euthanasia moments (2, 6, 18 weeks [W] and heart failure [HF]). Sham: operated control (blue); AoS: supravalvar aortic stenosis (red). (**A**) EF (%): left ventricle (LV) ejection fraction in percentage. (**B**) Meso (%): LV shortening of mesocardium in percentage. (**C**) LVPW: LV posterior wall shortening velocity. (**D**) E wave: early diastolic mitral inflow velocity. (**E**) A wave: late diastolic mitral inflow velocity. (**F**) E/A: ratio between E wave and A wave. Data are expressed as mean ± SD and analyzed by ANOVA and Tukey. *p* < 0.05. * AoS vs. respective Sham. Distinct letters signify statistical difference between moments; (*n* = 12–18 animals for each group).

Analysis of systolic function showed that ejection fraction (EF) decreased in AoS_HF_ compared to the other AoS groups; it was higher in AoS_6_ than Sham_6_ and lower in AoS_HF_ than Sham_HF_ (Figure 3A). Mesocardial shortening (Meso) was lower in AoS_HF_ than the other AoS groups; in AoS_18_ it was lower than AoS_6_ and AoS_2_. It was higher in AoS_6_ than Sham_6_ and lower in AoS_HF_ than Sham_HF_ (Figure 3B). Posterior wall shortening velocity (PWSV) values were lower in AoS_18_ and AoS_HF_ than their respective Shams (Figure 3C).

Analysis of diastolic function showed that E wave was higher in AoS_HF_ than the other AoS groups; it was more significant in AoS_6_ and AoS_18_ than AoS_2_. Early diastolic mitral inflow velocity (E wave) was higher in AoS_6_, AoS_18_, and AoS_HF_ compared to their respective Sham groups (Figure 3D). The late diastolic mitral inflow velocity (A wave) was smaller in AoS_HF_ than the other AoS groups; it was higher in AoS_6_ and AoS_18_ compared to the respective Shams and lower in AoS_HF_ than Sham_HF_ (Figure 3E). The E/A ratio was higher in AoS_HF_ compared to the other AoS groups; it was also higher in **AoS**_**HF**_ than **Sham**_**HF**_ (Figure 3F). Function analysis by echocardiogram showed that animals with aortic stenosis had early diastolic dysfunction which was accentuated with the evolution of RC and was accompanied by a decrease in systolic function in the HF phase.

### 2.4. Cardiac HIF-1α Expression

**Figure 4:**
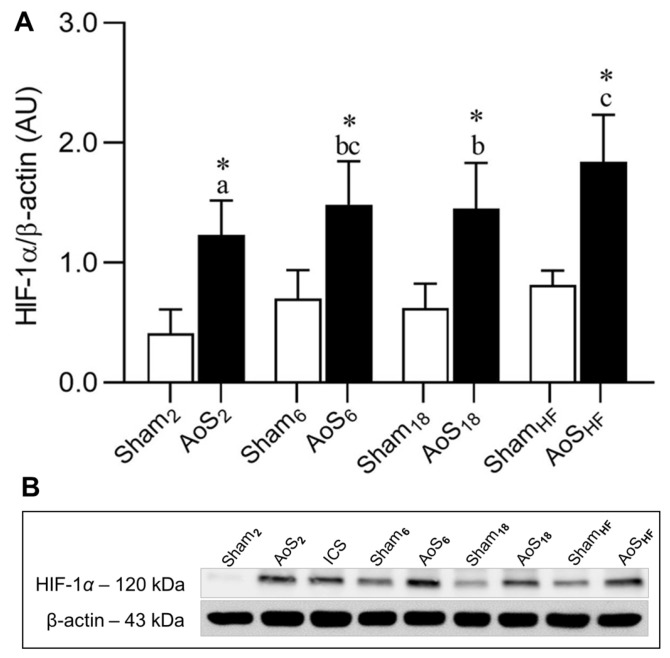
Hypoxia-inducible factor-1α (HIF-1α) protein expression evaluated by Western blot. (**A**) Quantification of HIF-1α. (**B**) Representative bands of protein. Sham: operated control; AoS: supravalvar aortic stenosis; HF: heart failure; Sham_2_: Sham 2 weeks; Sham_6_: Sham 6 weeks; Sham_18_: Sham 18 weeks; Sham_HF_: Sham HF; AoS_2_: AoS, 2 weeks; AoS_6_, 6 weeks; AoS_18_, 18 weeks; AoS_HF_, HF. ISC: Internal control of the sample; AU: arbitrary unity. Data are expressed as mean ± SD and analyzed by ANOVA and Tukey, *p* < 0.05. * AoS vs. respective Sham. Distinct letters signify statistical difference between moments (*n* = 8 animals for each group).

Analysis of evolution in animals with heart disease showed that HIF-1α was higher in AoS_HF_ than AoS_2_ and AoS_18_. The difference of 2.58% between AoS_6_ vs. AoS_HF_ (24.17%) and AoS_18_ vs. AoS_HF_ (26.76%) groups shows that, although there was a statistical difference in AoS_6_ and AoS_18_ vs. AoS_HF_, from a biological point of view their behavior seemed similar. There was a statistical difference in all AoS animals compared to their respective Shams (Figure 4). The increase in HIF-1α in AoS groups in relation to their respective controls may suggest myocardial hypoxia. Studies on the quantification of cardiac oxygen rate are necessary for further clarification.

### 2.5. Blood Glucose and Serum Insulin

**Figure 5:**
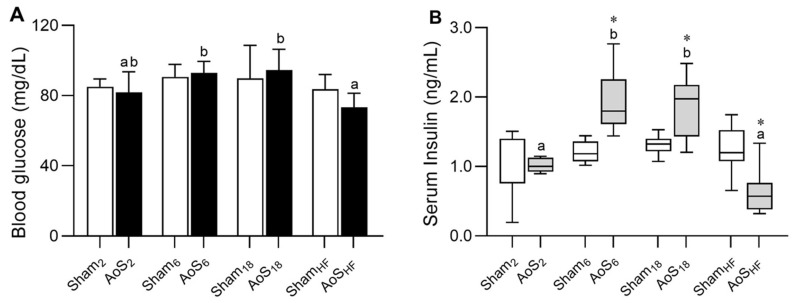
Glucose and insulin data. (**A**) Blood glucose. (**B**) Serum insulin. Group abbreviations are as defined in Figure 4. Data are expressed as mean ± SD and analyzed by ANOVA and Tukey in (**A**). Data expressed in median (Min and Max) and analyzed by ANOVANP and Dunn in (**B**). *p* < 0.05. * AoS vs. respective Sham. Distinct letters signify statistical difference between moments; (*n* = 8–12 animals for each group).

Glucose levels were lower in AoS_HF_ than AoS_6_ and AoS_18_, suggesting that time may interfere with the glycemic level (Figure 5A). Insulin levels increased in AoS_6_ and AoS_18_ compared to AoS_2_; however, they decreased in AoS_HF_ compared to AoS_6_ and AoS_18_. Insulin was higher in AoS_6_ and AoS_18_ than their respective Shams; it was significantly lower in AoS_HF_ compared to Sham_HF_ (Figure 5B). Insulin concentrations changed during the course of CR; the reduction in the AoS_HF_ phase may be due to the inefficiency in pancreatic cells to produce insulin.

### 2.6. Cardiac Insulin Signaling and Glucose Transporters 1 and 4

**Figure 6:**
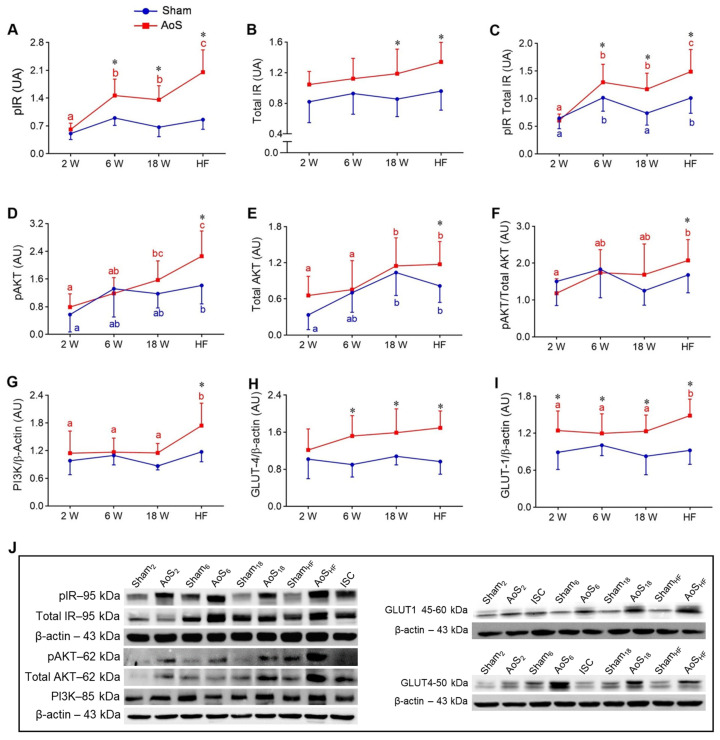
Cardiac insulin signaling and glucose transporters 1 and 4 at different euthanasia moments (2, 6, 18 weeks [W] and heart failure [HF]) evaluated by Western blot. Sham: operated control (blue); AoS: supravalvar aortic stenosis (red). Quantification of (**A**) pIR: phosphorylated insulin receptor, (**B**) Total IR: total insulin receptor, (**C**) pIR/Total IR: pIR/Total IR ratio, (**D**) pAKT: phosphorylated protein kinase B, (**E**) Total AKT: total protein kinase B, (**F**) pAKT/Total AKT: pAKT/Total AKT ratio; (**G**) PI3K: phosphoinositide 3-kinase, (**H**) GLUT4: glucose transporter 4, and (**I**) GLUT1: glucose transporter 1. (**J**) Representative bands of proteins. Data are expressed as mean ± SD and analyzed by ANOVA and Tukey. *p* < 0.05. * AoS vs. respective Sham. Distinct letters signify statistical difference between moments (*n* = 8 animals for each group).

With regard to phosphorylated insulin receptor (pIR) level, it was higher in AoS_HF_ than the other AoS groups, higher in AoS_18_ and AoS_6_ than AoS_2_, and more significant in AoS_HF_, AoS_18_, and AoS_6_ than their respective Shams (Figure 6A). Total insulin receptor (IR) increased in AoS_6_ and AoS_18_ compared to their respective Shams (Figure 6B). The pIR/Total IR ratio was higher in AoS_HF_ than the other AoS groups; in AoS_18_ and AoS_6_ it was higher than AoS_2_; it was higher in Sham_HF_ and Sham_6_ than Sham_18_ and Sham_2_. It was more significant in AoS_HF_, AoS_18_, and AoS_6_ than their respective Shams (Figure 6C). With regard to phosphorylated protein kinase B (pAKT), it was higher in AoS_HF_ than AoS_6_ and AoS_2_; higher in AoS_18_ and Sham_HF_ than AoS_2_; and it was also higher in AoS_HF_ than Sham_HF_ (Figure 6D). Total protein kinase B (AKT) values in AoS_HF_ and AoS_18_ were higher than AoS_6_ and AoS_2_; they were higher in Sham_HF_ and Sham_18_ than Sham_2_; and higher in AoS_HF_ than Sham_HF_ (Figure 6E). The pAKT/Total AKT ratio was higher in AoS_HF_ than AoS_2_; this was more significant in AoS_HF_ than Sham_HF_ (Figure 6F). Phosphoinositide 3-kinase (PI3K) was higher in AoS_HF_ than the other AoS groups; it was more significant in AoS_HF_ than Sham_HF_ (Figure 6G). Glucose transporters 4 (GLUT4) was higher in AoS_6_, AoS_18_, and AoS_HF_ compared to their respective Shams (Figure 6H). Glucose transporters 1 (GLUT1) was higher in AoS_HF_ than the other AoS groups; it was more significant in AoS groups then their respective Shams (Figure 6I). These results show activation of the insulin pathway and increased GLUT1 expression, accentuated in the heart failure phase. These changes, which could result in increased glucose uptake, are aimed at maintaining hypertrophied heart energy demand. Studies to quantify cardiac glucose uptake are necessary for further clarification.

### 2.7. Cardiac Glycolysis and Glycogen

**Figure 7:**
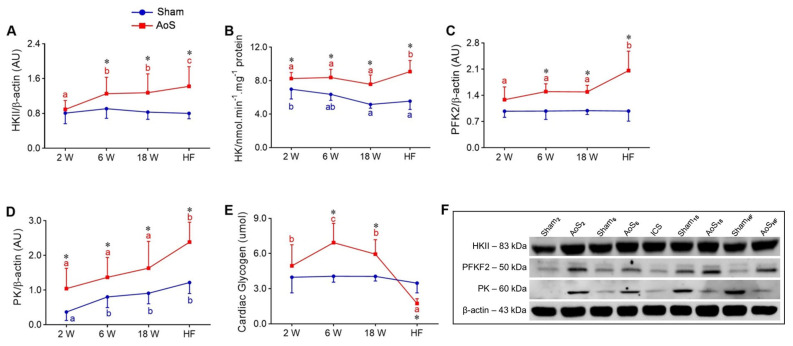
Myocardial glycolysis and glycogen at different moments of euthanasia (2, 6, 18 weeks [W] and heart failure [HF]) evaluated by Western blot and enzymatic activity assay. Sham: operated control (blue); AoS: supravalvar aortic stenosis (red). Quantification of (**A**) HKII: hexokinase II, (**B**): HK: activity of hexokinase II, (**C**) PFKB2: phosphofruct kinase 2, (**D**) PK: pyruvate kinase, and (**E**): cardiac glycogen. (**F**) Representative bands of proteins. Data are expressed as mean ± SD and analyzed by ANOVA and Tukey. *p* < 0.05. * AoS vs. respective Sham. Distinct letters signify statistical difference between moments; (*n* = 8 animals for each group).

Hexokinase II (HKII) was higher in AoS_HF_ than the other AoS animals; it was higher in AoS_18_ and AoS_6_ than AoS_2_. It was also more significant in AoS_HF_, AoS_18_, and AoS_6_ than their respective Shams (Figure 7A). HKII activity was higher in AoS_HF_ than the other AoS groups; it was also lower in Sham_18_ and Sham_6_ than Sham_2_. It was also more significant in all AoS groups than their respective Shams (Figure 7B). 6-phosphofructo-2-kinase/fructose-2,6-biphosphatase 2 (PFK2) was higher in AoS_HF_ than the other AoS groups; it was higher in AoS_6_, AoS_18,_ and AoS_HF_ than their respective Shams (Figure 7C). Pyruvate kinase (PK) was higher in AoS_HF_ than the other AoS groups; it was lower in Sham_2_ than the other control groups. PK was higher in all AoS groups than their respective Shams (Figure 7D). Over time, cardiac glycogen exhibited lower concentration in AoS_HF_ than AoS_2_, AoS_6_, and AoS_18_; concentration was elevated in AoS_6_ compared to AoS_2_ and AoS_18_. Cardiac glycogen was higher in AoS_6_ and AoS_18_ and lower in AoS_HF_ than their respective Shams (Figure 7E). Generally, the glycolysis pathway increases in HF to increase energy for the heart. In the most severe phase of the disease, glycogen stores are reduced, either by insufficient storage or by greater use of this substrate than exogenous glucose.

### 2.8. Cardiac Aerobic and Anaerobic Pathway

**Figure 8:**
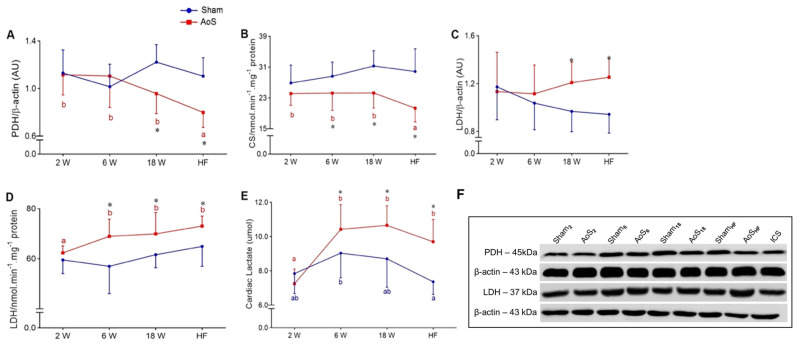
Myocardial aerobic and anaerobic metabolism at different euthanasia moments (2, 6, 18 weeks [W] and heart failure [HF]) evaluated by Western blot and enzymatic activity assay. Sham: operated control (blue); AoS: supravalvar aortic stenosis (red). Quantification of (**A**) PDH: pyruvate dehydrogenase, (**B**) CS: citrate synthase activity, (**C**) LDH: lactate dehydrogenase, (**D**) LDH: LDH activity, and (**E**) cardiac lactate concentration. (**F**) Representative bands of proteins. Data are expressed as mean ± SD and analyzed by ANOVA and Tukey. *p* < 0.05. * AoS vs. respective Sham. Distinct letters signify statistical difference between moments; (*n* = 8 animals for each group).

Pyruvate dehydrogenase (PDH) expression was lower in AoS_HF_ than the other AoS animals; it was lower in AoS_HF_ and AoS_18_ than their respective Shams (Figure 8A). Citrate synthase (CS) activity was lower in AoS_HF_ than the other AoS animals; it was lower in AoS_6_, AoS_18_, and AoS_HF_ compared to their respective Shams (Figure 8B). Lactate dehydrogenase (LDH) expression increased in AoS_HF_ and AoS_18_ than in their respective Shams (Figure 8C). LDH activity was more significant in AoS_6_, AoS_18_, and AoS_HF_ than AoS_2_; it was more significant in AoS_6_, AoS_18_, and AoS_HF_ than their respective Shams (Figure 8D). Lactate levels were more significant in AoS_6_, AoS_18_, and AoS_HF_ than AoS_2_; they was higher in Sham_6_ than Sham_HF_. They were also more significant in AoS_6_, AoS_18_, and AoS_HF_ than their respective Shams (Figure 8E). Our results show deviation from the aerobic to anaerobic pathway in hypertrophied myocardium of rats with AoS. The data associated with the increase in HIF-1α suggests an O_2_ deficit in cardiac tissue that arises during the remodeling process.

**Figure 9:**
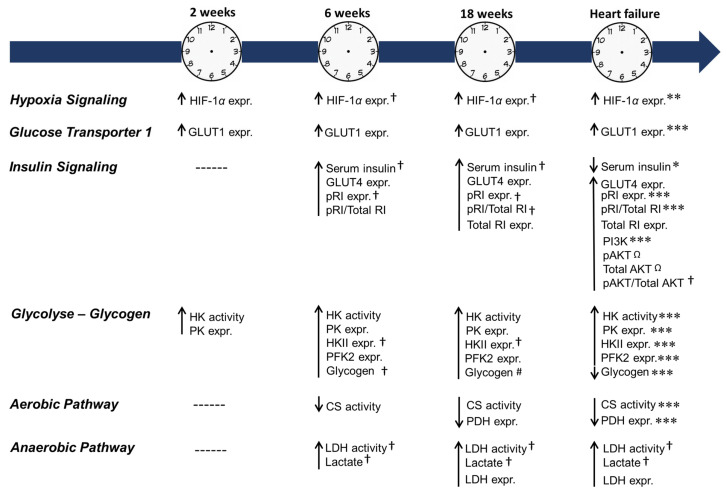
Overview of results observed during the evolution process of cardiac remodeling from hypertrophy to heart failure. Expr.: expression. Protein abbreviations are as defined in Figure 4, Figure 5, Figure 6, Figure 7 and Figure 8. Different symbols mean statistical difference between AoS vs. Sham (**↑** increase or ↓ decrease) or between the AoS groups († vs. AoS**_2_**; # vs. Aos**_6_**; *** vs. Aos**_2, 6, 18_**; ** vs. Aos **_2, 18_**; * vs. Aos**_6, 18_**; Ω vs. Aos**_2, 6_**), *p* < 0.05.

Figure 9 shows the overview results obtained from HIF-1α and glucose metabolism during the evolution process of cardiac remodeling due to pressure overload.

### 2.9. Canonical Correlation Analysis (CCA)

**Figure 10:**
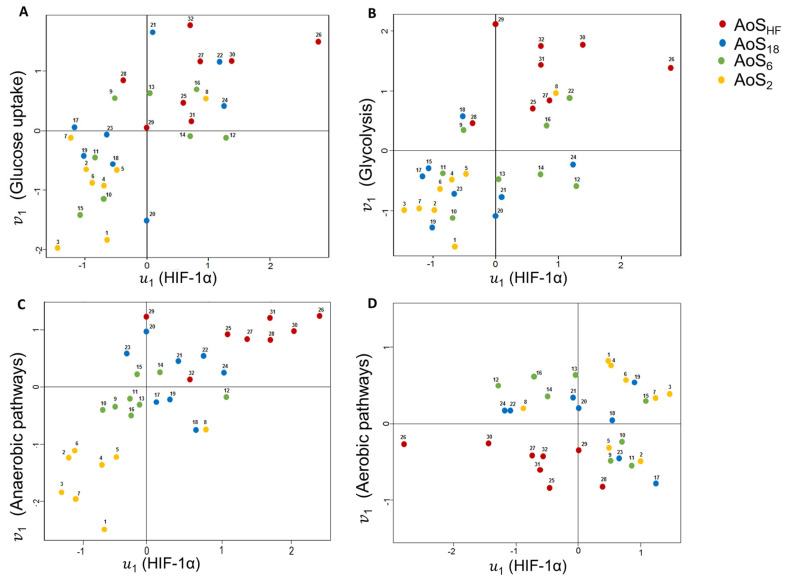
Scatter plot of canonical variables in aortic stenosis groups. AoS: supravalvar aortic stenosis; HF: heart failure; • AoS_2_, 2 weeks; • AoS_6_, 6 weeks; • AoS_18_, 18 weeks; • AoS_HF_, HF; HIF-1α: factor inducible hypoxia. (**A**) Insulin signaling and glucose transporters 1 and 4 (total and phosphorylated insulin receptor, total and phosphorylated protein kinase B, phosphoinositide 3-kinase and glucose transporter 1 and 4 expression); *p* < 0.027. (**B**) Glycolysis (hexokinase activity, hexokinase II, phosphofruct kinase 2 and pyruvate kinase expression); *p* < 0.018. (**C**) Anaerobic pathways (lactate dehydrogenase expression and activity); *p* < 0.024. (**D**) Aerobic pathways (pyruvate dehydrogenase expression and citrate synthase activity); *p* < 0.957. Canonical correlations: insulin signaling and glucose transporters 1 and 4, 0.671; glycolysis, 0.625; anaerobic pathways, 0.675; aerobic pathways, 0.054. 𝑢1 = 1.00 (HIF-1α). Insulin signaling and glucose transporters 1 and 4: A𝑣1 = 0.40 pIR + 0.23 total IR + 0.56 pAKT − 0.01 total AKT + 0.54 PI3K + 0.74 GLUT1 + 0.26 GLUT4. Glycolysis: A𝑣1 = 0.32 HKII + 0.66 HK + 0.81 PFK2 + 0.28 PK. Anaerobic pathways: A𝑣1 = 0.45 LDH − 0.99 LDH activity. Aerobic pathways: A𝑣1 = 0.789 PDH + 0.723 LDH.

The primary objective of CCA is to measure a linear association between sets of variables. In this study, we measured the association between HIF-1α and the following ensemble derived from (a) insulin signaling and glucose transporters 1 and 4 (pIR, Total IR, pAKT, Total AKT, PI3K, GLUT1, and GLUT4 expressions; (b) glycolysis (HKII activity and HKII, PFK2 and PK expressions); (c) anaerobic pathway (LDH activity and LDH expression); and (d) aerobic pathway (CS activity and PDH expression). There was a positive linear association between canonical variable 𝑢1 (HIF-1α) and 𝑣1 (Figure 10A–C). In this sense, 𝑢1 presents a high association with 𝑣1, mainly in the AoS_HF_ group, and a low association in the AoS_2_ group. AoS_6_ and AoS_18_ animals have dispersed behavior around the average. There was no significant association between HIF-1α and the aerobic pathway parameters (Figure 10D).

## 3. Discussion

This study analyzed HIF-1α, the glucose metabolism, aerobic and anaerobic pathways, and the association between HIF-1α and variables during the transition from compensated hypertrophy to HF. To our knowledge, this is the first study to show heart disease progression regarding this major hypoxia marker and glucose metabolism.

We found that HIF-1α increased in all groups with heart disease, with an important increase during CR progression, especially in the HF phase. High levels of HIF-1α, resulting from a decrease in its degradation in the presence of tissue hypoxia [5,20,21,22], may be due to the imbalance between the supply and consumption of O_2_ by the myocardium due to disharmony in the proliferative capacity of the capillary network, vascular rarefaction, the degree of concentric hypertrophy, and myocardial fibrosis [23,24,25,26,27]. The accentuation seen over time of these myocardial structural changes may have been responsible for increasing HIF-1α in the different phases of aortic stenosis.

Although HIF-1α has a cardioprotective action by restoring energy homeostasis [22,28], there is evidence that persistent elevation, as observed in the AoS_6_, AoS_18_ and AoS_HF_ groups, may participate in the genesis of hypertrophy [12] along with other factors such as parietal mechanical stress and the neuroendocrine system [27,29,30,31,32]. In addition to action in the hypertrophic process, HIF-1α can also participate in the genesis of ventricular dysfunction observed in cardiac groups, as it causes a suppression of/decrease in sarcoplasmic/endoplasmic reticulum Ca^2+^ATPase 2a (SERCA2a) synthesis [33,34] or fibrosis development [35,36]. Other factors may also have participated in this dysfunction, such as apoptosis, reactivation of fetal gene expression, altered sarcomere structure, insufficient angiogenesis, mitochondrial dysfunction, impairment of the cellular Ca^2+^ transient, and metabolic reprogramming [26,27,31,37]. Although there are investigations that have shown increased HIF-1α in hypertrophy related to heart failure [11], to our knowledge no other studies have systematically evaluated HIF-1α behavior during the different phases of cardiac remodeling evolution due to overload mechanics.

GLUT1 increased in all groups with pressure overload which was accentuated during cardiac remodeling, mainly in the HF phase. GLUT1 is a glucose transporter, independent of insulin, responsible for glucose uptake in the fetal stage [38]. Although its expression reduces after birth, GLUT1 can be reinduced in cardiac patients [39]. The early rise in GLUT1 indicates an attempt to increase glucose uptake by the myocardium from the initial aggression phase (AoS_2_). As HIF-1α is a transcription factor for GLUT1 [28,40], and it increased during the evolutionary process, we can suggest that HIF-1α may be involved in the GLUT1 elevation observed in this study. Other potential factors implied in increasing this transporter are hypertrophic stimuli, such as alpha-adrenergic agonist and ischemia/hypoxia [38,41]. Our results are consistent with the literature, which shows elevated GLUT1 during pathological hypertrophy [27,38,42].

Insulin signaling pathway activity rose in all aortic stenosis groups from the sixth week onwards, increasing over time with the exception of AoS_HF_ which showed a drop in insulin level. Hyperinsulinemia in AoS_6_ and AoS_18_, consistent with insulin resistance in cardiac patients [15,43], aims to maintain glucose homeostasis at the expense of elevating this hormone. Increased insulin may have promoted a rise in IR phosphorylation but was not accompanied by an elevation in main insulin signaling pathway proteins, PI3K and AKT. Once that this cascade had been compromised in the cardiac groups, other factors might have increased sarcolemmal GLUT4, such as ischemia and cardiac contraction [38]. In AoS_HF_, in contrast to AoS_6_ and AoS_18_ groups, the hypoinsulinemia may have occurred from the pancreas being unable to produce insulin probably due to structural and functional changes and decreased food intake in animals with HF [44]. In hypoinsulinemia, increased expression of pIR, Total IR, PI3K, pAKT, and Total AKT may be aimed at maintaining adequate glucose uptake for the hypertrophied myocardium to attenuate energy dysregulation in an attempt to prolong cell survival. The mechanisms responsible for the increase in glucose uptake proteins in hypoinsulinemia in heart failure need to be clarified. According to the literature, in pathological hypertrophy, GLUT4 levels are reduced [27,38,42] or increased [45]; our results are in agreement with Cook and collaborators [45] as we found a rise in and stabilization of GLUT4 after the sixth week of aortic stenosis. With regard to the insulin/IR/PI3K/AKT signaling pathway, the literature shows an increase in these variables in pressure-overload-induced cardiac animals [37], corroborating our findings, except for insulin in the HF phase.

The glycolytic pathway showed an increase in all cardiopathy groups; this intensified during remodeling and was more expressive in the HF period. AoS_2_ animals, in which left ventricle (LV) hypertrophy was less pronounced than the other AoS groups, showed increased HKII activity and PK expression. HKII enzyme phosphorylates glucose into glucose 6 phosphate (G-6P) to prevent glucose release from inside the cell. Its activity is inhibited by high G-6P and ATP concentrations and stimulated by high levels of adenosine diphosphate, adenosine monophosphate, and phosphate [46]; therefore, the presumed increase in these factors by hypoxia in our study could have caused a rise in HKII enzyme activity in all cardiopathy groups.

PK is responsible for producing pyruvate and ATP from the phosphoenolpyruvate substrate [47]. Different factors are involved in the increased expression of this enzyme, e.g., HIF-1α [48]; since this transcriptional factor is high in AoS_2_, it could be responsible for raising PK expression. In AoS_6_, AoS_18_, and AoS_HF_, in addition to the changes shown in AoS_2_, there were novel alterations, such as increased HKII and PFK2 expression, possibly due to greater energy demand; the factors involved in raising these glycolytic enzymes may be HIF-1α and insulin [49,50]. These two elements might have participated until the 18th week; since there was a decay in insulin in the HF phase, HIF-1α may have been largely responsible for these enzyme changes. Intensification of the glycolytic pathway throughout CR progression, mainly in the HF phase, displays an attempt to compensate for the increasing energy deficit resulting from the decline in O_2_ supply. According to the literature, glycolysis is elevated in different animal models of cardiac hypertrophy [51], corroborating our results. However, to our knowledge, no study has evaluated the glycolytic pathway during CR progression due to pressure overload.

Although glycolysis was exacerbated, an increase in cardiac glycogen storage was seen in AoS_6_ and AoS_18_ and a robust reduction seen in AoS**_HF_** animals. This elevation signals that glycogen synthesis was more significant than glycogenolysis, probably due to the increase in exogenous glucose uptake by the increased translocation of GLUT 1 and 4 (Figure 6H,I) and glycogen synthase kinase 3β (GSK-3β) inhibition [31]. During CR progression, the glycogen decay after the 18th week, which intensified in the HF phase, indicates that hypertrophied hearts started to preferentially use glucose from glycogen stores as opposed to exogenous glucose; this is possibly due to increased energy demand and reduced insulin or enzyme activity involved in glycogenesis [52]. According to the literature, glycogen synthesis is elevated in hypertrophied hearts, corroborating our findings [53]. We did not find any study that assessed myocardial glycogen levels in severe heart failure.

The anaerobic and aerobic pathways changed in cardiopathy groups from the sixth week onwards with an increase in LDH activity and lactate concentration, and reduced CS activity; also, LDH expression rose in AoS_18_ and PDH expression decreased in AoS_HF_. These modifications indicate a shift from the aerobic to anaerobic pathway, possibly by tissue hypoxia, i.e., lack of myocardial O_2_ due to mitochondrial dysfunction and disharmony between the capillary network and degree of hypertrophy [17,54,55]. This deviation towards the anaerobic pathway suggests that although there was an increase in glycolysis, it may not have been accompanied by a rise in glucose oxidation [38]. Interestingly, although the aerobic and anaerobic pathways displayed concomitant changes in the sixth week, during evolution of the hypertrophic process, the anaerobic route remained stable. In contrast, the aerobic pathway showed a marked decline in the HF phase. Our findings agree with data from the literature observed in different cardiac hypertrophy models [53,56]. To our knowledge, there is no information regarding the moment when the aerobic pathway changes to anaerobic during CR progression due to pressure overload.

The canonical association data revealed that HIF-1α had a positive and significant linear correlation with the sets of variables that analyzed insulin signaling and GLUT 1 and GLUT 4, glycolysis, and the anaerobic pathway. The most intense correlations from each group occurred with proteins that are expressed by transcriptional factor HIF-1α, such as GLUT1, PFK2, and LDH [11,28,57]. Although these associations do not indicate a cause-and-effect relationship, their significance shows that HIF-1α may be involved in gene alterations during the process of glycidic metabolic remodeling in this experimental model. These results have biological support in the literature, where HIF-1α is described as participating in the transcription of proteins involved in glucose metabolism. We suggest further studies that perform HIF-1α blocking to understand the cause-and-effect relationship of this protein and its action on the metabolism and function of the remodeled heart. This and other concerns observed in this work can only be obtained through additional investigation into HIF-1α, energy metabolism, and heart remodeling.

## 4. Materials and Methods

### 4.1. Animals, Supravalvar Surgery, and Experimental Groups

Twenty-one-day-old male Wistar rats were housed in individual cages with controlled temperature (24 ± 2 °C), humidity (55 ± 5%), and light (12 h light/dark cycle), and free access to food and water. All experimental procedures were performed according to the Guide for the Care and Use of Laboratory Animals published by the National Research Council (2011) and approved by the Ethics Committee on Animals Experiments of the Botucatu Medical School, São Paulo State University, UNESP (1081/2014-CEUA).

Supravalvular aortic stenosis (AoS) in young rats has been used as an experimental model to study chronic HF, initially with preserved ejection fraction which may progress to cardiac systolic insufficiency [23,58,59,60]. Ventricular dysfunction occurs gradually, mimicking the development of heart disease in humans. AoS was induced surgically as previously described [23,61,62]. Briefly, a silver clip (0.60 mm internal diameter) was placed on the ascending aorta approximately 3 mm from its root (the AoS group; *n* = 56). Sham rats underwent the same surgery but without aortic banding (*n* = 54). Animals from both groups were evaluated at 2, 6, and 18 weeks after surgery and when they presented signs of severe HF, 28–30th week [63,64]. Mortality percentages were as follows: AoS_2_ (0%), AoS_6_ (17%), AoS_18_ (27%), AoS_HF_ (36%). There were no deaths in the Sham group. The following subgroups were formed: Sham_2_ (*n* = 12), Sham_6_ (*n* = 12), Sham_18_ (*n* = 15), Sham_HF_ (*n* = 15), AoS_2_ (*n* = 12), AoS_6_ (*n* = 14), AoS_18_ (*n* = 16), and AoS_HF_ (*n* = 14). Rats were observed daily to identify clinical signs of HF: tachypnea, apathetic behavior, altered hair, and loss of muscle mass. The diagnosis was pathologically confirmed post mortem by analyzing the presence and magnitude [(−) absent; (+) light; (++) moderate; (+++) severe] of the following signs: RV hypertrophy, left atrial thrombus, pleural effusion, hemorrhagic liver, and ascites [23]. Animals in the AoS_HF_ group presented all the signs that characterized severe HF. At the end of each experimental period (2, 6, 18 weeks and HF), animals were fasted for 8 h and were anesthetized with a mixture of ketamine (50 mg/kg, intraperitoneal [i.p.]) and xylazine (10 mg/kg, i.p.) and euthanized by decapitation.

### 4.2. Body and Lung Weights and Post Mortem Cardiac Morphology

The final body weight (BW), lung weight, and lung/tibia ratio were measured at the end of each experimental period. Cardiac hypertrophy was determined by macroscopic analysis of the following parameters: atrium (AT), LV, and RV weights, as well as their ratio with tibia length.

### 4.3. Structural and Functional Echocardiographic Data

Echocardiogram was performed before each euthanasia using a commercially available echocardiograph (General Electric Medical Systems, Vivid S6, Tirat Carmel, Israel) equipped with a 5–11.5 MHz multifrequency transducer as previously described [23,58,59,61]. The following structural variables were analyzed: LA/AO, LVDD, LVWDT, and RWT. The following parameters assessed LV function: percentage of Meso, percentage of EF, PWSV, E and A waves, and E/A ratio.

### 4.4. Serum Glucose and Insulin Levels

Blood samples were collected from the tail tip before euthanasia for glucose analysis and were assessed using a handheld glucometer (Accu-Chek Go Kit; Roche Diagnostic Brazil Ltd., São Paulo, Brazil). Fasting serum insulin concentrations were measured in blood samples collected after euthanasia and centrifuged at 1620× *g* for 10 min at 4 °C using an enzyme-linked immunosorbent assay (ELISA) kit (EMD Millipore Corporation, Billerica, MA, USA), according to manufacturer instructions.

### 4.5. Cardiac HIF-1α and Glucose Metabolism

Cardiac expression of HIF-1α and the proteins involved in glucose metabolism were evaluated by Western blot. LV tissue samples were homogenized in cold RIPA lysis buffer (Amresco, Solon, OH, USA) containing protease (Sigma-Aldrich, St. Louis, MO, USA) and phosphatase (Roche Diagnostics, Indianapolis, IN, USA) inhibitors. The homogenate was centrifuged at 12,000× *g* for 20 min at 4 °C and supernatant collected. For the extraction of GLUT1 and GLUT4, protein membranes were homogenized in extraction buffer containing 1M Tris-HCl, 25 mM pentetic acid (DTPA), β-Mercaptoethanol, 0.1 M Phenylmethylsulfonyl Fluoride (PMSF), and protease inhibitor. The homogenate was centrifuged at 1000× *g* for 3 min at 4 °C to remove insoluble material. The supernatant was then centrifuged at 18,000× *g* for 10 min at 4 °C and transferred to an ultracentrifuge (Sorvall WX80 Ultracentrifuge—Thermo Scientific, Waltham, MA, USA) at 100,000× *g* for 45 min at 4 °C. After ultracentrifugation, the pellet was resuspended in 100 μL of cold RIPA lysis buffer. Protein concentrations were determined using a Pierce BCA Protein Assay kit (Thermo Scientific, Wilmington, DE, USA). Samples (50 μg) were subjected to SDS-PAGE in polyacrylamide gels (6% or 10% depending on protein molecular weight). After electrophoresis, proteins were electrotransferred to nitrocellulose membrane (Armsham Biosciences, Piscataway, NJ, USA). The blotted membranes were blocked with 5% nonfat dry milk in Tris-buffered saline/Tween-20 (25 mM Tris, pH 7.5, 140 mM sodium chloride, 3mM potassium chloride, and 0.1% Tween-20) for 2 h at room temperature. Membranes were then incubated overnight at 4 °C–8 °C with primary antibody against: HIF-1α (1:500; Abcam, Cambridge, MA, USA; #ab463); IR (1:1000; Cell Signaling, Danvers, MA, USA; #3025); pIR (Tyr1150/1151; 1:1000; Cell Signaling; #3024); PI3K (1:1000; Cell Signaling; #4292); AKT (1:500; Santa Cruz, Delaware Ave, Albany, NY, USA; # sc-5298); pAKT (Thr 308; 1:500; Santa Cruz; #sc-16646-R); HKII (1:1000; Cell Signaling; #2106); PFK2 (1:1000; Millipore, Temecula, CA, USA; #07-1530); PK (1:1000; Cell Signaling; #3186); LDH (1:1000; Cell Signaling; #2012); PDH (1:1000; Cell Signaling; #2784); GLUT1 (1:1000; Cell Signaling; #12939); and GLUT4 (1:1000; Cell Signaling; #2213). Primary antibody binding was detected using peroxidase-conjugated secondary antibodies (anti-mouse or anti-rabbit IgG, depending on the protein; 1:5000–1:10,000, Abcam; #ab97023 or #ab97080) incubated for 1.5 h at room temperature. A control rat was present on all gels at the standard internal sample control (ISC). Protein bands were visualized via chemiluminescent detection (Supersignal, Pierce, Rockford, IL, USA) in a Western blot detection system (ImageQuant™ LAS 4000—GE Healthcare Life Sciences, Chalfont, UK), and quantified by densitometry using Image J analysis software version 1.52. Targeted bands were normalized to the expression of cardiac β-actin (1:1000; Cell Signaling; #4967).

### 4.6. Enzymatic Activity of Cardiac Glucose Metabolism

Activities of key glucose metabolism enzymes were analyzed, including HKII, LDH, and CS. Heart LV samples were homogenized 1:20 (wt/vol) in 50mM Tris-HCl, 1mM EDTA, and protease inhibitor cocktail, pH 7.4 using a Polytron instrument (Kinematica, Littau-Lucerne, Switzerland). The lysate was centrifuged at 12,000 rpm for 10 min at 4 °C, and the supernatant was collected. All enzyme activities were determined at 25 °C using a Spectra Max 250 microplate spectrophotometer (Molecular Devices, Sunnyvale, CA, USA), and assay buffer without sample was used as blank. HKII assay medium consisted of 75 mM Tris-HCl, 7.5mM MgCl_2_, 0.8 mM EDTA, 1.5 mM KCl, 4 mM 2 mercaptoethanol, 0.4 mM NADP^+^, 2.5 mM ATP, 1 mM glucose, 1.4 units glucose-6-phosphate dehydrogenase, and 0.05% Triton X-100. Total assay volume was 165 µL, and 10 µL of homogenate was added. The assay was initiated by adding glucose [65]. LDH was assayed in a reaction mix containing 20 mM Tris, 6.0 mM pyruvate, and 5.0 mM NADH and monitored at 340 nm. CS assay medium consisted of 50 mM Tris-HCl, 1 mM EDTA, 0.2 mM DTNB, 0.1 mM acetyl-CoA, 0.5 mM oxaloacetate, and 0.05% Triton X-100. The total assay volume was 165 uL, and 10 uL of homogenate was added. The assay was initiated by adding oxaloacetate [66]. The rate of change in absorbance was monitored at 412 nm (ε = 13.6 μmol × mL^−1^ × cm^−1^).

### 4.7. Cardiac Glycogen

Cardiac glycogen content was determined as described by Trinder (1969) [67]. The test medium consisted of 30 mM phosphate buffer, 1 mM phenol, 12.5U glucose oxidase, 0.8U peroxidase, 0.29 mM 4-aminoantipyrine, and 0.05% Triton X-100. Total assay volume was 165 µL, and 20 µL of homogenate was added. Absorbance delta was monitored at 505 nm. Corrected values were subtracted from the control well reading (without amyloglucosidase) and from the reaction well reading (with amyloglucosidase) for each sample. Glucose content was determined using a glucose standard.

### 4.8. Statistical Analysis

Data are expressed as mean ± SD (standard deviation) or median (minimum [Min] and maximum [Max] values). Echocardiographic, cardiac metabolic, and structural variables, considering both sources of variation, i.e., AoS (absence or presence) and euthanasia moment (2, 6, 18 weeks and HF) were analyzed by ANOVA using a 2 × 4 factorial scheme with a completely randomized design; this was complemented with Tukey’s multiple comparisons test (for variables with adherence normality) and non-parametric analysis of variance technique (ANOVANP) followed by Dunn’s post hoc test (for variables with a lack of adherence normality). The choice of parametric or non-parametric procedure was based on the Kolmogorov–Smirnov normality test [68]. Regarding the study of the linear association between HIF-1 and the set of metabolic variables, the exploratory multivariate data technique involving the canonical correlation was used [69]. All statistical conclusions were discussed at a 5% significance level. The level of significance considered was 5%. Analyses were performed using RStudio version 1.1.442 (Boston, MA, USA), and graphics were generated using GraphPad Prism 8 (GraphPad Software Inc., San Diego, CA, USA).

## 5. Conclusions

In conclusion, our hypothesis was confirmed as there was an early change and intensification of the glucose metabolism, observed by increased activity and/or expression of glucose transporters, glycolysis enzymes, and proteins involved in lactate production and the intracellular insulin signaling cascade, concomitant with high levels of HIF-1α during the progression from hypertrophy to heart failure.

## Figures and Tables

**Table 1:** Clinical and pathological data of heart failure (HF).

Signs of HF	Grups	Intensity and RF
Respiratory pattern change	Sham_HF_	(−)
	AoS_HF_	(+++) 100%
Ascites	Sham_HF_	(−)
	AoS_HF_	(+++) 100%
Pleural effusion	Sham_HF_	(−)
	AoS_HF_	(+++) 100%
Thrombus in LA	Sham_HF_	(−)
	AoS_HF_	(++) 20%; (+++) 80%
Hypertrophy of RV	Sham_HF_	(−)
	AoS_HF_	(++) 10%; (+++) 90%

RF: relative frequency in percentage (%); intensity of heart failure signs: (−) absent; (++) moderate; (+++) severe; LA: left atrium; RV: right ventricle; Sham_HF_: operated control (*n* = 15); AoS_HF_: AoS with severe heart failure (*n* = 14).

## Data Availability

All data were included in this manuscript. Additional datasets generated during and/or analyzed during the current study are available from the corresponding author upon request.

## References

[1] Ritterhoff J., Tian R. (2017). Metabolism in Cardiomyopathy: Every Substrate Matters. Cardiovasc. Res..

[2] Gibb A.A., Hill B.G. (2018). Metabolic Coordination of Physiological and Pathological Cardiac Remodeling. Circ. Res..

[3] Karwi Q.G., Uddin G.M., Ho K.L., Lopaschuk G.D. (2018). Loss of Metabolic Flexibility in the Failing Heart. Front. Cardiovasc. Med..

[4] Felker G.M., Mann D.L. (2015). Heart Failure: A Companion to Braunwald’s Heart Disease.

[5] Rocha S. (2007). Gene Regulation under Low Oxygen: Holding Your Breath for Transcription. Trends Biochem. Sci..

[6] Semenza G.L. (2001). Hypoxia-Inducible Factor 1: Oxygen Homeostasis and Disease Pathophysiology. Trends Mol. Med..

[7] Sutton M.G.S.J., Plappert T., Hilpisch K.E., Abraham W.T., Hayes D.L., Chinchoy E. (2006). Sustained Reverse Left Ventricular Structural Remodeling with Cardiac Resynchronization at One Year Is a Function of Etiology: Quantitative Doppler Echocardiographic Evidence from the Multicenter InSync Randomized Clinical Evaluation (MIRACLE). Circulation.

[8] Ye J., Gao Z., Yin J., He Q. (2007). Hypoxia Is a Potential Risk Factor for Chronic Inflammation and Adiponectin Reduction in Adipose Tissue of *Ob*/*Ob* and Dietary Obese Mice. Am. J. Physiol. Endocrinol. Metab..

[9] Lim C.S., Kiriakidis S., Sandison A., Paleolog E.M., Davies A.H. (2013). Hypoxia-Inducible Factor Pathway and Diseases of the Vascular Wall. J. Vasc. Surg..

[10] Semenza G.L. (2009). Involvement of Oxygen-Sensing Pathways in Physiologic and Pathologic Erythropoiesis. Blood.

[11] Li X., Zhang Q., Nasser M., Xu L., Zhang X., Zhu P., He Q., Zhao M. (2020). Oxygen Homeostasis and Cardiovascular Disease: A Role for HIF?. Biomed. Pharmacother..

[12] Mirtschink P., Krek W. (2016). Hypoxia-Driven Glycolytic and Fructolytic Metabolic Programs: Pivotal to Hypertrophic Heart Disease. Biochim. Biophys. Acta.

[13] Kolwicz S.C., Tian R. (2011). Glucose Metabolism and Cardiac Hypertrophy. Cardiovasc. Res..

[14] Doenst T., Nguyen T.D., Abel E.D. (2013). Cardiac Metabolism in Heart Failure: Implications beyond ATP Production. Circ. Res..

[15] Zhang L., Jaswal J.S., Ussher J.R., Sankaralingam S., Wagg C., Zaugg M., Lopaschuk G.D. (2013). Cardiac Insulin-Resistance and Decreased Mitochondrial Energy Production Precede the Development of Systolic Heart Failure after Pressure-Overload Hypertrophy. Circ. Heart Fail..

[16] Doenst T., Pytel G., Schrepper A., Amorim P., Färber G., Shingu Y., Mohr F.W., Schwarzer M. (2010). Decreased Rates of Substrate Oxidation Ex Vivo Predict the Onset of Heart Failure and Contractile Dysfunction in Rats with Pressure Overload. Cardiovasc. Res..

[17] Peterzan M.A., Lygate C.A., Neubauer S., Rider O.J. (2017). Metabolic Remodeling in Hypertrophied and Failing Myocardium: A Review. Am. J. Physiol. Heart Circ. Physiol..

[18] Lazzeroni D., Rimoldi O., Camici P.G. (2016). From Left Ventricular Hypertrophy to Dysfunction and Failure. Circ. J..

[19] Friehs I., Moran A.M., Stamm C., Colan S.D., Takeuchi K., Cao-Danh H., Rader C.M., McGowan F.X., del Nido P.J. (1999). Impaired Glucose Transporter Activity in Pressure-Overload Hypertrophy Is an Early Indicator of Progression to Failure. Circulation.

[20] Wigerup C., Påhlman S., Bexell D. (2016). Therapeutic Targeting of Hypoxia and Hypoxia-Inducible Factors in Cancer. Pharmacol. Ther..

[21] Semenza G.L. (2012). Hypoxia-Inducible Factors: Mediators of Cancer Progression and Targets for Cancer Therapy. Trends Pharmacol. Sci..

[22] Masoud G.N., Li W. (2015). HIF-1α Pathway: Role, Regulation and Intervention for Cancer Therapy. Acta Pharm. Sin. B.

[23] Sant’Ana P.G., Batah S.S., Leão P.S., Teodoro W.R., de Souza S.L.B., Ferreira Mota G.A., Vileigas D.F., da Silva V.L., de Campos D.H.S., Okoshi K. (2018). Heart Remodeling Produced by Aortic Stenosis Promotes Cardiomyocyte Apoptosis Mediated by Collagen V Imbalance. Pathophysiology.

[24] Tromp J., Khan M.A.F., Klip I.T., Meyer S., de Boer R.A., Jaarsma T., Hillege H., van Veldhuisen D.J., van der Meer P., Voors A.A. (2017). Biomarker Profiles in Heart Failure Patients with Preserved and Reduced Ejection Fraction. J. Am. Heart Assoc..

[25] Mohammed S.F., Hussain S., Mirzoyev S.A., Edwards W.D., Maleszewski J.J., Redfield M.M. (2015). Coronary Microvascular Rarefaction and Myocardial Fibrosis in Heart Failure with Preserved Ejection Fraction. Circulation.

[26] Oldfield C.J., Duhamel T.A., Dhalla N.S. (2020). Mechanisms for the Transition from Physiological to Pathological Cardiac Hypertrophy. Can. J. Physiol. Pharmacol..

[27] Nakamura M., Sadoshima J. (2018). Mechanisms of Physiological and Pathological Cardiac Hypertrophy. Nat. Rev. Cardiol..

[28] Hurst J.H. (2016). William Kaelin, Peter Ratcliffe, and Gregg Semenza Receive the 2016 Albert Lasker Basic Medical Research Award. J. Clin. Investig..

[29] Anzai T. (2018). Inflammatory Mechanisms of Cardiovascular Remodeling. Circ. J..

[30] Pitoulis F.G., Terracciano C.M. (2020). Heart Plasticity in Response to Pressure- and Volume-Overload: A Review of Findings in Compensated and Decompensated Phenotypes. Front. Physiol..

[31] Riehle C., Abel E.D. (2016). Insulin Signaling and Heart Failure. Circ. Res..

[32] Cohn J.N., Ferrari R., Sharpe N. (2000). Cardiac Remodeling--Concepts and Clinical Implications: A Consensus Paper from an International Forum on Cardiac Remodeling. Behalf of an International Forum on Cardiac Remodeling. J. Am. Coll. Cardiol..

[33] Bekeredjian R., Walton C.B., MacCannell K.A., Ecker J., Kruse F., Outten J.T., Sutcliffe D., Gerard R.D., Bruick R.K., Shohet R.V. (2010). Conditional HIF-1alpha Expression Produces a Reversible Cardiomyopathy. PLoS ONE.

[34] Ronkainen V.-P., Skoumal R., Tavi P. (2011). Hypoxia and HIF-1 Suppress SERCA2a Expression in Embryonic Cardiac Myocytes through Two Interdependent Hypoxia Response Elements. J. Mol. Cell. Cardiol..

[35] Bourdier G., Détrait M., Bouyon S., Lemarié E., Brasseur S., Doutreleau S., Pépin J.-L., Godin-Ribuot D., Belaidi E., Arnaud C. (2020). Intermittent Hypoxia Triggers Early Cardiac Remodeling and Contractile Dysfunction in the Time-Course of Ischemic Cardiomyopathy in Rats. J. Am. Heart Assoc..

[36] Ruthenborg R.J., Ban J.-J., Wazir A., Takeda N., Kim J.-W. (2014). Regulation of Wound Healing and Fibrosis by Hypoxia and Hypoxia-Inducible Factor-1. Mol. Cells.

[37] Shimizu I., Minamino T., Toko H., Okada S., Ikeda H., Yasuda N., Tateno K., Moriya J., Yokoyama M., Nojima A. (2010). Excessive Cardiac Insulin Signaling Exacerbates Systolic Dysfunction Induced by Pressure Overload in Rodents. J. Clin. Investig..

[38] Shao D., Tian R. (2015). Glucose Transporters in Cardiac Metabolism and Hypertrophy. Compr. Physiol..

[39] Pereira R.O., Wende A.R., Olsen C., Soto J., Rawlings T., Zhu Y., Anderson S.M., Abel E.D. (2013). Inducible Overexpression of GLUT1 Prevents Mitochondrial Dysfunction and Attenuates Structural Remodeling in Pressure Overload but Does Not Prevent Left Ventricular Dysfunction. J. Am. Heart Assoc..

[40] Semenza G. (2002). Signal Transduction to Hypoxia-Inducible Factor 1. Biochem. Pharmacol..

[41] Montessuit C., Thorburn A. (1999). Transcriptional Activation of the Glucose Transporter GLUT1 in Ventricular Cardiac Myocytes by Hypertrophic Agonists. J. Biol. Chem..

[42] Tham Y.K., Bernardo B.C., Ooi J.Y.Y., Weeks K.L., McMullen J.R. (2015). Pathophysiology of Cardiac Hypertrophy and Heart Failure: Signaling Pathways and Novel Therapeutic Targets. Arch. Toxicol..

[43] Ascaso J.F., Real J.T., Priego A., Carmena R., Romero P., Valdecabres C. (2001). Cuantificación de Insulinorresistencia Con Los Valores de Insulina Basal e Índice HOMA En Una Población No Diabética. Med. Clin..

[44] DeBoer M.D. (2009). Animal Models of Anorexia and Cachexia. Expert Opin. Drug Discov..

[45] Cook S.A., Varela-Carver A., Mongillo M., Kleinert C., Khan M.T., Leccisotti L., Strickland N., Matsui T., Das S., Rosenzweig A. (2010). Abnormal Myocardial Insulin Signalling in Type 2 Diabetes and Left-Ventricular Dysfunction. Eur. Heart J..

[46] Katz A.M. (2011). Physiology of the Heart.

[47] Li X.-B., Gu J.-D., Zhou Q.-H. (2015). Review of Aerobic Glycolysis and Its Key Enzymes—New Targets for Lung Cancer Therapy. Thorac. Cancer.

[48] Alves-Filho J.C., Pålsson-McDermott E.M. (2016). Pyruvate Kinase M2: A Potential Target for Regulating Inflammation. Front. Immunol..

[49] Wilson J.E. (2003). Isozymes of Mammalian Hexokinase: Structure, Subcellular Localization and Metabolic Function. J. Exp. Biol..

[50] Mor I., Cheung E.C., Vousden K.H. (2011). Control of Glycolysis through Regulation of PFK1: Old Friends and Recent Additions. Cold Spring Harb. Symp. Quant. Biol..

[51] Tian R., Musi N., D’Agostino J., Hirshman M.F., Goodyear L.J. (2001). Increased Adenosine Monophosphate-Activated Protein Kinase Activity in Rat Hearts with Pressure-Overload Hypertrophy. Circulation.

[52] Schaefer S., Ramasamy R. (1997). Glycogen Utilization and Ischemic Injury in the Isolated Rat Heart. Cardiovasc. Res..

[53] Tran D.H., Wang Z.V. (2019). Glucose Metabolism in Cardiac Hypertrophy and Heart Failure. J. Am. Heart Assoc..

[54] Rabinowitz J.D., Enerbäck S. (2020). Lactate: The Ugly Duckling of Energy Metabolism. Nat. Metab..

[55] Urbańska K., Orzechowski A. (2019). Unappreciated Role of LDHA and LDHB to Control Apoptosis and Autophagy in Tumor Cells. Int. J. Mol. Sci..

[56] Taegtmeyer H., Overturf M.L. (1988). Effects of Moderate Hypertension on Cardiac Function and Metabolism in the Rabbit. Hypertension.

[57] Sousa Fialho M.D.L., Abd Jamil A.H., Stannard G.A., Heather L.C. (2019). Hypoxia-Inducible Factor 1 Signalling, Metabolism and Its Therapeutic Potential in Cardiovascular Disease. Biochim. Biophys. Acta Mol. Basis. Dis..

[58] Mota G.A.F., de Souza S.L.B., da Silva V.L., Gatto M., de Campos D.H.S., Sant’Ana P.G., Vileigas D.F., Padovani C.R., Casarini D.E., de Oliveira E.M. (2020). Cardioprotection Generated by Aerobic Exercise Training Is Not Related to the Proliferation of Cardiomyocytes and Angiotensin-(1-7) Levels in the Hearts of Rats with Supravalvar Aortic Stenosis. Cell. Physiol. Biochem..

[59] De Souza S.L.B., Mota G.A.F., da Silva V.L., Sant’Ana P.G., Vileigas D.F., de Campos D.H.S., Padovani C.R., Rodrigues M.A.M., do Nascimento A.F., Sugizaki M.M. (2020). Adjustments in β-Adrenergic Signaling Contribute to the Amelioration of Cardiac Dysfunction by Exercise Training in Supravalvular Aortic Stenosis. Cell. Physiol. Biochem..

[60] Mazeto I.F.S., Okoshi K., Silveira C.F.S.M.P., Sant’Ana P.G., da Silva V.L., Mota G.A.F., de Souza S.L.B., Vileigas D.F., Padovani C.R., Cicogna A.C. (2021). Calcium Homeostasis Behavior and Cardiac Function on Left Ventricular Remodeling by Pressure Overload. Braz. J. Med. Biol. Res..

[61] Casquel De Tomasi L., Salomé Campos D.H., Grippa Sant’Ana P., Okoshi K., Padovani C.R., Masahiro Murata G., Nguyen S., Kolwicz S.C., Cicogna A.C. (2018). Pathological Hypertrophy and Cardiac Dysfunction Are Linked to Aberrant Endogenous Unsaturated Fatty Acid Metabolism. PLoS ONE.

[62] Souza R.W.A., Piedade W.P., Soares L.C., Souza P.A.T., Aguiar A.F., Vechetti-Júnior I.J., Campos D.H.S., Fernandes A.A.H., Okoshi K., Carvalho R.F. (2014). Aerobic Exercise Training Prevents Heart Failure-Induced Skeletal Muscle Atrophy by Anti-Catabolic, but Not Anabolic Actions. PLoS ONE.

[63] Momken I., Kahapip J., Bahi L., Badoual T., Hittinger L., Ventura-Clapier R., Veksler V. (2003). Does Angiotensin-Converting Enzyme Inhibition Improve the Energetic Status of Cardiac and Skeletal Muscles in Heart Failure Induced by Aortic Stenosis in Rats?. J. Mol. Cell. Cardiol..

[64] Moreira V.O., de Castro A.V.B., Yaegaschi M.Y., Cicogna A.C., Okoshi M.P., Pereira C.A., Aragon F.F., Bruno M.B., Padovani C.R., Okoshi K. (2006). Echocardiographic Criteria for the Definition of Ventricular Dysfunction Severity in Aortic Banded Rats. Arq. Bras. Cardiol..

[65] Crabtree B., Newsholme E.A. (1972). The Activities of Phosphorylase, Hexokinase, Phosphofructokinase, Lactate Dehydrogenase and the Glycerol 3-Phosphate Dehydrogenases in Muscles from Vertebrates and Invertebrates. Biochem. J..

[66] Alp P.R., Newsholme E.A., Zammit V.A. (1976). Activities of Citrate Synthase and NAD+-Linked and NADP+-Linked Isocitrate Dehydrogenase in Muscle from Vertebrates and Invertebrates. Biochem. J..

[67] Trinder P. (1969). Determination of Blood Glucose Using an Oxidase-Peroxidase System with a Non-Carcinogenic Chromogen. J. Clin. Pathol..

[68] Zar J.H. (2010). Bioestatical Analysis.

[69] Mingoti S.M. (2005). Análise de Dados Através de Métodos de Estatística Multivariada: Uma Abordagem Aplicada.

